# Preferences and Attitudes of Cardiologists in Management of Patients with Cancer

**DOI:** 10.1089/pmr.2022.0014

**Published:** 2022-11-21

**Authors:** Ibrahim Azar, Stephani Wang, Vikram Dhillon, Jacqueline Kenitz, Dawn Lombardo, Roderick Deano, Syed Mahmood, Hirva Mamdani, Anthony F. Shields, Philip Agop Philip, Michael Stellini, Joshua Schulman-Marcus

**Affiliations:** ^1^ Division of Hematology-Oncology, Department of Medicine, Karmanos Cancer Institute, Wayne State University, Detroit, Michigan, USA.; ^2^ IHA Hematology Oncology, Pontiac, Michigan, USA.; ^3^ Division of Cardiology, Department of Medicine, University of California, Irvine, California, USA.; ^4^ Division of Cardiology, Department of Medicine, Albany Medical College, Albany, New York, USA.; ^5^ Division of Cardiology, Department of Medicine, University of Wisconsin, Madison, Wisconsin, USA.; ^6^ Division of Cardiology, Department of Medicine, Weill Cornell Medical Center, New York, New York, USA.; ^7^ Division of Palliative Care, Department of Medicine, Wayne State University and Karmanos Cancer Institute, Detroit, Michigan, USA.

**Keywords:** cardio-oncology, high value care, physician communication

## Abstract

**Background:**

With recent improvements in survival of cancer patients and common use of high-value care at end of life, the management of cardiovascular disease (CVD) in patients with cancer is increasingly important. To our knowledge, there are no current U.S. data examining how the presence and extent of cancer influence cardiologists' decision making for common cardiovascular conditions.

**Methods:**

An anonymous online vignette-based survey of cardiologists was conducted at five U.S. institutions investigating how the extent of gastrointestinal and thoracic malignancies (prior/localized, metastatic) would influence treatment recommendations for atrial fibrillation (AF), aortic stenosis, unstable angina (UA), and obstructive coronary artery disease (CAD).

**Results:**

Thirty-three percent (86/259) of cardiologists completed the survey between September and November 2019. Participants were 67% male, 51% below age 40, and 58% had five or more years of clinical experience. Majority of cardiologists practiced at teaching hospitals (72%) and were noninterventional (63%). Cardiologists were more likely to recommend procedural interventions for patients with localized cancer than for those with metastatic disease: AF (left atrial appendage occlusion: 20% vs. 8%), atrial stenosis (aortic valve repair: 83% vs. 11%), UA (left heart catheter: 70% vs. 27%), and obstructive CAD (percutaneous coronary intervention: 81% vs. 38%). In patients with metastatic cancer, most cardiologists sought an oncology (82%) or a palliative care (69%) consultation. However, a persistent trend of undertreatment in patients with localized cancers and overtreatment in patients with end-of-life disease was apparent.

**Conclusions:**

Cardiologists were less likely to recommend invasive cardiovascular therapies to patients with metastatic cancer. This preference pattern likely reflects the influence of comorbidities and quality of life expectation on cardiologists' treatment recommendations but may also be related to the stigma of advanced cancer. Better communication between cardiologists and oncologists is necessary to provide a personalized care of patients with cancer and CVD that would maximize treatment benefit with least morbidity.

## Background

Cardiovascular disease (CVD) and cancer are the two most prevalent causes of morbidity and mortality in the United States of America.^1,2^ With significant improvements in cancer treatment for the past two decades, the population of cancer survivors is increasing.^3^ Owing to shared risk factors between CVD and cancer, cancer survivors are experiencing an increased incidence of CVD, and the burden of CVD is becoming a pressing concern.^4^ In addition, systemic therapies and radiation can carry increased cardiotoxicities, compounding the problem. For instance, trastuzumab, an anti-HER2 monoclonal antibody used in breast and gastric cancer, is associated with myocardial hibernation leading to reduced left ventricular ejection fraction, cardiomyopathy, and congestive heart failure.^5^

Advances in oncology have accelerated exponentially recently, and new drugs in oncology are being approved by FDA at a rapid rate, outpacing every other medical specialty.^6^ This has challenged the ability of oncologists to keep up with the literature with approvals coming in monthly. Although the life expectancy of patients afflicted by some malignancies have changed dramatically for the better (e.g., chronic myeloid leukemia and lung cancer with identifiable molecular drivers with small molecule inhibitors), prognosis remains grim for other malignancies (e.g., pancreatic cancer). Data on the knowledge of cardiologists in cancer prognostication are lacking. A recent survey from the European Society of Cardiology shows that >85% of European cardiologists acknowledge the need for more education on cardio-oncology.^7^

Currently, there are no developed societal guidelines to guide which cardiac interventions are appropriate for patients battling different stages of cancer. In delivering high-value care to these patients, data suggest that close collaboration between cardiologists and oncologists can lead to improved outcomes and may benefit from the creation of multidisciplinary teams with a focus on cardio-oncology.^8,9^ For example, early involvement of cardiologists in patients with atrial fibrillation (AF) and cancer has been associated with increased prescriptions for DOACs and an overall reduction in major adverse cardiac events.^10^ To the best of our knowledge, there are no studies to date evaluating the implicit biases present in clinical decision making of cardiologists for management of common cardiovascular complications in patients with localized and metastatic cancers in the United States.

Treatment biases become more pertinent to recognize in patients with metastatic cancer because all potential treatment decisions need to be made after weighing in the palliative nature of the treatment of the underlying disease. Patients with a limited life expectancy frequently get overtreated and a significant portion of medical care provided (admissions, procedures, laboratory, and imaging) becomes futile or nonbeneficial in nature.^11^ For instance, prescribing aspirin for secondary prevention in a patient with a primary pancreatic cancer, where the median survival time is less than six months, would provide minimal benefit to the patient and could complicate their care.^12^

Similar interventions may be associated with patient harm, poor quality of life (QoL), add to cost of care and inappropriate resource utilization.^13^ Recognizing when active cancer treatment is becoming nonbeneficial also has a major benefit of reducing suffering and maintaining the dignity of patient's suffering from advanced cancers. Involving palliative care specialists in the care of patients with metastatic disease also requires better understanding of cultural sensitivities. Several multidisciplinary projects such as The Dignity Project have focused on refining communication styles to include families in goals of care decision making without violating a patient's autonomy.^14^

The overall objective of this study was to assess how the presence of cancer influences treatment decisions by a cardiologist. More specifically, we aimed to understand how frequently cardiologists sought aggressive treatment in localized and metastatic cancer and if palliative services or oncology were consulted.

## Methods

We conducted a multicenter anonymous online survey of cardiologists and cardiology fellows working in the community or at academic centers to assess how the presence and extent of cancer might influence treatment recommendations. The survey was conducted at five U.S institutions: Albany Medical College, University of California, Irvine, University of Wisconsin, Wayne State University, and Weill Cornell Medical Center. Demographic data were collected and presented in Table 1. After providing demographic information, participants were presented with a series of three clinical vignettes designed to elicit an intervention from the cardiologist (Table 2, Supplementary Tables S1 and S2).

**Table 1.** Demographic Characteristics of Cardiologists

Participation	
Responders	33% (86)
Nonresponders	67% (173)
Gender	
Men	66.3% (57)
Women	33.7% (29)
Age (years)	
Mean	36.5
Median	34
Experience (years)	
Mean	15
Median	8
Practice setting	
Teaching hospital	62% (53)
Community	24% (33)
Subspecialty	Responders
Noninterventional cardiologists	62.8% (54)
Interventional cardiologists	8.1% (7)
EP	11.6% (10)
Advanced HF cardiologist	9.3% (8)
Cardio-oncology specialist	1.2% (1)
Others	7% (6)

EP, electrophysiologists; HF, heart failure.

**Table 2.** Clinical Vignette #2 Aortic Stenosis

A 70-year-old woman with history of stage 2 adenocarcinoma of the colon underwent resection two years ago and currently is in remission. She currently presents with increasing shortness of breath with activity and lower extremity swelling for the past 2 weeks. A transthoracic echocardiogram shows that her left ventricular function is 45% with an aortic valve area of 0.8 cm^2^ (previously 1.2 cm^2^ 12 months ago). Cardiac catheterization does not show significant coronary disease requiring intervention but reveals a mean aortic gradient of 54 mmHg. Please rank-order your treatment recommendation (one is most preferred and five least preferred)? • Medical therapy with possible BAV • Transcatheter aortic valve replacement • Surgical aortic valve replacement • Refer to palliative care • Oncology consultation before making final decision.
• The same patient presented above has an active stage III lung cancer and is under treatment.
• The same patient presented above has diffusely metastatic pancreatic cancer.

BAV, balloon valvuloplasty.

Participants were presented with three common cardiovascular complications: AF in the setting of prior GI bleeding, symptomatic severe aortic stenosis (AS), and unstable angina (UA). Following each vignette, participants were asked to rank-order 4–5 treatment recommendations in terms of preference. Each vignette had modifiers for tumor extent and asked the cardiologist if their preference would change for the same patient with localized cancer and then with metastatic cancer (for instance, nonsmall cell, pancreatic).

For purposes of this analysis, we defined a “preferred” treatment recommendation as an option ranked first or second. All responses were collected and analyzed through a Qualtrics platform. The study was approved by the Albany Medical College Institutional Review Board.

## Results

Among 259 cardiologists at five institutions who were recruited, 86 (33%) completed the survey between September and November 2019 (Table 1). Participants were 67% male with a median age of 30 for men and 35 for women, 51% below age 40, and 58% had five or more years of clinical experience. A majority practiced at a teaching hospital (72%); 54 noninterventional cardiologists (63%), 10 electrophysiologists, and 7 interventional cardiologists. Table 1 summarizes the demographics.

**FIG. 1.**
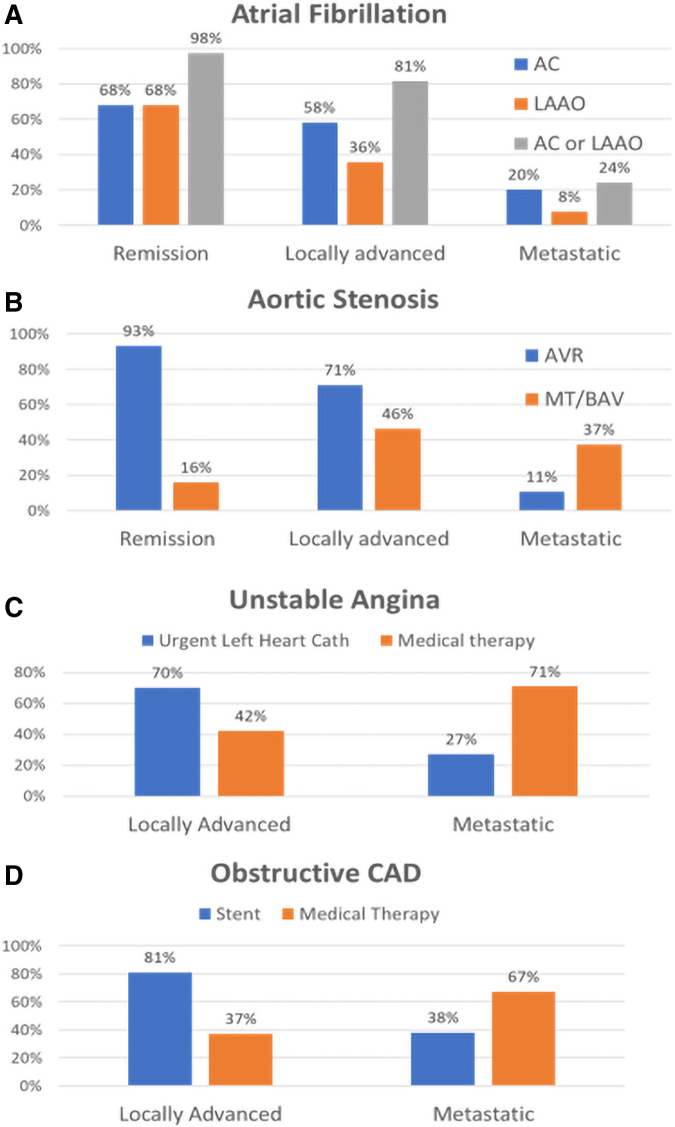
Graphical representation of cardiologists' management preferences in common cardiovascular conditions.

In the AF vignette (Table 3A; Fig. 1A), almost all (98%) cardiologists recommended either anticoagulation or left atrial appendage occlusion device (LAAO, e.g., Watchman) for patients with colon cancer in remission. In contrast, 81% recommended the same interventions for locally advanced NSCLC receiving treatment, whereas a quarter of cardiologists (24%) still recommended anticoagulation (AC) or LAAO for patients with diffusely metastatic pancreatic cancer. Of these, only six cardiologists recommended inserting a LAAO in the metastatic setting. Cardiologists were likely to recommend a palliative care consultation in 11% of patients with locally advanced cancer and 65% with metastatic disease. Almost half (∼48%) of cardiologists asked oncologists to weigh in on patients with cancer in remission, versus 83% of patients with locally advanced and 71% in patients with metastatic disease.

**Table 3.** Cardiologists' Management Preferences in Common Cardiovascular Conditions

A. AF	Remission	Locally advanced	Metastatic
Response (*n*)	81	81	79
Anticoagulation	68% (55)	58% (47)	20% (16)
Watchman	68% (55)	36% (29)	8% (6)
AC or watchman	98% (79)	81% (66)	24% (19)
No anticoagulation	16% (13)	12% (10)	37% (29)
Palliative care consult	0	11% (9)	65% (51)
Oncology consult	48% (39)	83% (67)	71% (56)

**B. AS**	**Remission**	**Locally advanced**	**Metastatic**
Response (*n*)	75	69	75
Med/BAV	16% (12)	46% (32)	37% (28)
TAVR	92% (69)	71% (49)	9% (7)
SAVR	49% (37)	3% (2)	1% (1)
Any valve	93% (70)	71% (49)	11% (8)
Palliative care consult	0%	16% (11)	73% (55)
Oncology consult	43% (32)	81% (56)	79% (59)

**C. Angina**	**Locally advanced**	**Metastatic**	
Response (*n*)	71	73	
Urgent cardiac cath	70% (50)	27% (20)	
Medical therapy	42% (30)	71% (52)	
Cardiac CT	14% (10)	8% (6)	
Oncology consult	73% (52)	93% (68)	

**D. Obstructive CAD**	**Locally advanced**	**Metastatic**	
Stent	73	69	
Medical therapy	37% (27)	67% (46)	
Bare metal stent	49% (36)	16% (11)	
Drug eluting stent	52% (38)	29% (20)	
Any stent	81% (59)	38% (26)	
Oncology consult	62% (45)	88% (61)	

AC, anticoagulation; AF, atrial fibrillation; AS, aortic stenosis; CAD, coronary artery disease; CT, computer tomography; SAVR, surgical aortic valve replacement; TAVR, transcatheter aortic valve replacement.

In the AS vignette, valve replacement (transcatheter or surgical) was the preferred recommendation for 93% of patients with prior cancer and 71% with locally advanced cancer under current treatment. Only eight cardiologists (11%) recommended valve placement in the setting of aggressive metastatic disease (with only one surgically placed). Medical therapy with balloon valvuloplasty was chosen by 46% of cardiologists in localized and 37% in metastatic cancers. Palliative care consultation was only selected 16% of the time by cardiologists in locally advanced disease versus 73% in metastatic setting. Four in five cardiologists consulted oncology in locally advanced and metastatic settings (81% and 79%, respectively).

In the UA vignette, urgent cardiac catheterization was a preferred recommendation for locally advanced cancer, whereas medical therapy was recommended for metastatic disease. In the patients with node-positive NSCLC, 70% of cardiologists chose an urgent left catheterization for UA. With metastatic NSCLC, 27% chose urgent catheterization. Reassuringly, 93% of cardiologists would obtain an oncology consultation in the metastatic setting.

If the UA patient had already been found to have obstructive coronary disease, stenting was the preferred recommendation for a patient with locally advanced cancer in 81% of the patients, whereas medical therapy was selected for 67% of the patients with metastatic disease. The percentage of cardiologists willing to perform a stent increased to 38% in the presence of coronary artery disease (CAD) in metastatic NSCLC. A majority of cardiologists preferred a consultation with oncology before making treatment decisions for patients with CAD and cancer (62% locally advanced and 88% metastatic).

## Discussion

Although there is increasing interest in cardio-oncology, there are few data or guidelines to inform the management of common cardiology conditions in patients with malignancy.^15^ Patients with active cancer or reduced expected lifespan have often been excluded from trials of advanced cardiac therapies. The degree to which they derive similar benefits compared with patients without cancer is usually unclear. The shortfall in data is likely to be more acutely felt as medical advances increase the duration of progression-free survival for many types of metastatic cancer.

The practice patterns of American cardiologists toward patients with cancer has not been established. We herein presented the first report of preferences of U.S. cardiologists regarding patients with cancer. In this multicenter vignette-based survey, we found that cardiologists were less likely to recommend aggressive cardiovascular therapies to patients with aggressive metastatic cancer. Instead, such patients were more likely to be recommended medical therapy. Most cardiologists reported consulting oncology before decision making in patients with cancer and CVD, with the majority reaching out to palliative care in aggressive metastatic disease.

Bringing high-value care to oncology patients affected by CVD is complicated to navigate. There is a great degree of heterogeneity among different malignancies and survival can vary greatly from one cancer to the other (e.g., metastatic pancreatic cancer vs. CLL). This inherent ambiguity was purposefully reflected in our vignettes. In the UA and obstructive CAD vignettes, the locally advanced scenario mentions lymph node involvement that encompasses stages IIB to IIC with an associated five-year OS ranging from 53% to 13%.^16^ The metastatic scenario also did not mention whether NSCLC had a molecular driver, which would impact survival greatly. For example, ALK-positive metastatic lung cancer survivors have a life expectancy of about seven years.^17^

In this light, it is reassuring that most cardiologists would reach out to the patient's oncologist before making decisions. Ideally, the oncology referrals reported in this survey from a low of 43% for AS for cancer in remission to 93% for angina in metastatic disease would all be close to 100%. Whether the reason for oncology consultations is to determine prognosis of the patients or to discuss the co-management of complex cardio-oncology case (e.g., myelosuppressive chemotherapy with dual antiplatelet therapy and/or timing of procedures while immunosuppressed) was not studied. Greater education and multidisciplinary collaboration will be increasingly necessary to deliver the best cardiac care to the growing number of patients living with cancer. The formation of dedicated cardio-oncology services should be the standard for tertiary and quaternary centers.

Our survey results suggest cardiologists will appropriately recommend less invasive therapies for patients with aggressive metastatic disease. However, our results indicate a preference by some cardiologists toward interventions and treatments that may have no benefit in patients with metastatic disease that represent continued overutilization of resources and wasteful management. For example, one in four cardiologists would recommend treatment of AF in a metastatic pancreatic cancer with expected survival of less than a year and 1 in 12 would recommend proceeding with inserting a LAAO device. The majority of cardiologists recommended a palliative care consultation for patients with metastatic disease (65% for AF and 73% for AS).

This preference pattern likely reflects a reasonable tendency to modify recommendations based on comorbidities and expected QoL. The increased involvement of palliative care is a recognition that treatment decisions are more nuanced, require a thorough conversation with the patient, understanding their ultimate goals for the treatment being offered and personalizing therapies or interventions on a case-by-case basis. In too many corners of medicine, stigma is still associated with palliative care and some physicians only consult palliative care for hospice services. Studies from the United States and China have shown that early involvement of palliative care in patients with metastatic lung and esophageal cancer increased survival and improved QoL.^18,19^ Our survey did not ask to specify the reason behind the palliative care consultation, but it is hoped that greater education of physicians would lead to near-total involvement of palliative care consultations for patients with metastatic disease.

Cancer patients suffer from perceived health-related stigma. Whether cancer survivors and cancer patients with good prognosis are undertreated by cardiologists is a topic of debate. Observational studies have suggested a tendency toward underutilization of guideline-directed therapies for patients with cancer who have myocardial infarction.^20^ However, these differences may also reflect many patient-specific and cancer-specific factors. Recently, several observational studies have reported that patients with severe AS and cancer who undergo aortic valve replacement (surgical or transcatheter) have similar short-term outcomes compared with patients without cancer and improved survival compared with patients with cancer managed medically.^21–23^

In our study, reassuringly >90% cardiologist recommended standard of care treatments for patients with cancers in remission for AF (94% watchman or anticoagulation) and AS (98% transcatheter aortic valve replacement [TAVR] or surgical aortic valve replacement [SAVR]). One limitation of our survey is the lack of a vignette of a noncancer patient to assess for undertreatment. Historical controls reveal a small level of stigma and undertreatment. In the UA scenario, patients with localized disease should receive a left heart catheterization (LHC), and historically, cardiologists recommend ∼80%–85% of the general population for urgent LHC. In our survey, only 70% of the cardiologists preferred an LHC in localize disease. Another limitation of our survey is that the majority of responders were noninterventional cardiologist and the results may not be generalizable to interventional cardiologists. Our cohort was in addition quite young and whether a difference in perspective on cancer outcomes based on age and experience exists has not been evaluated due to low numbers.

As cancer treatments evolve constantly, cardiotoxicities are better delineated and cardiovascular techniques evolve, increasingly complex cardiac care for cancer patients and survivors will come to the forefront of routine clinical practice. Better communication between cardiologists and oncologists and increased involvement of palliative care will be key to deliver high-value care to these patients.

## Supplementary Material

Supplemental data

Supplemental data

## Abbreviations Used

AC: anticoagulation
AF: atrial fibrillation
AS: aortic stenosis
BAV: balloon valvuloplasty
CAD: coronary artery disease
CT: computer tomography
CVD: cardiovascular disease
EP: electrophysiologist
HF: heart failure
LHC: left heart catheterization
MACE: major adverse cardiac events
QoL: quality of life
SAVR: surgical aortic valve replacement
TAVR: transcatheter aortic valve replacement
UA: unstable angina
