# Supplementary figures and images for: Preferences and Attitudes of Cardiologists in Management of Patients with Cancer
Source: Palliat Med Rep. 2022 Nov 21;3(1):279–86. doi: 10.1089/pmr.2022.0014 (PMC9712055; doi:10.1089/pmr.2022.0014)

Supplementary Table 1: Atrial fibrillation vignette


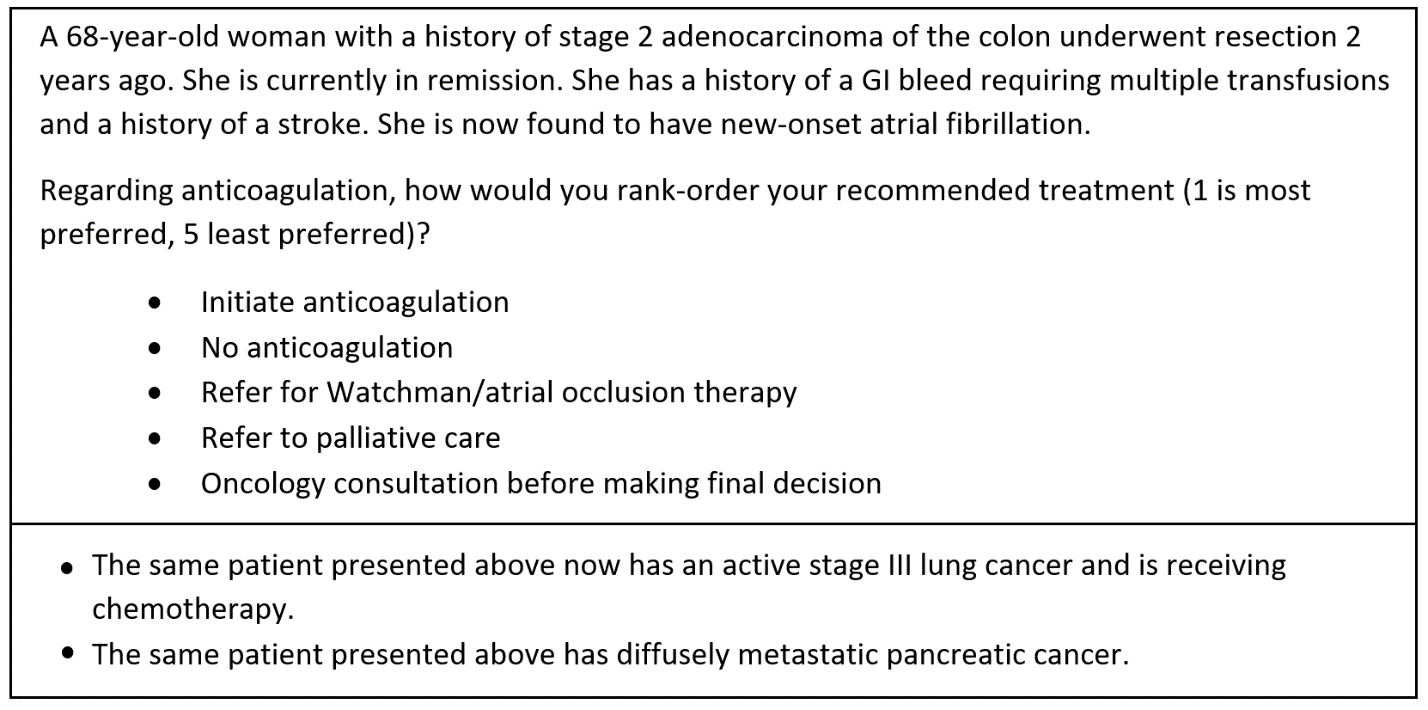

Supplement: Supplemental data [file Suppl_TableS1.docx]

Supplementary Table 2: Unstable angina vignette


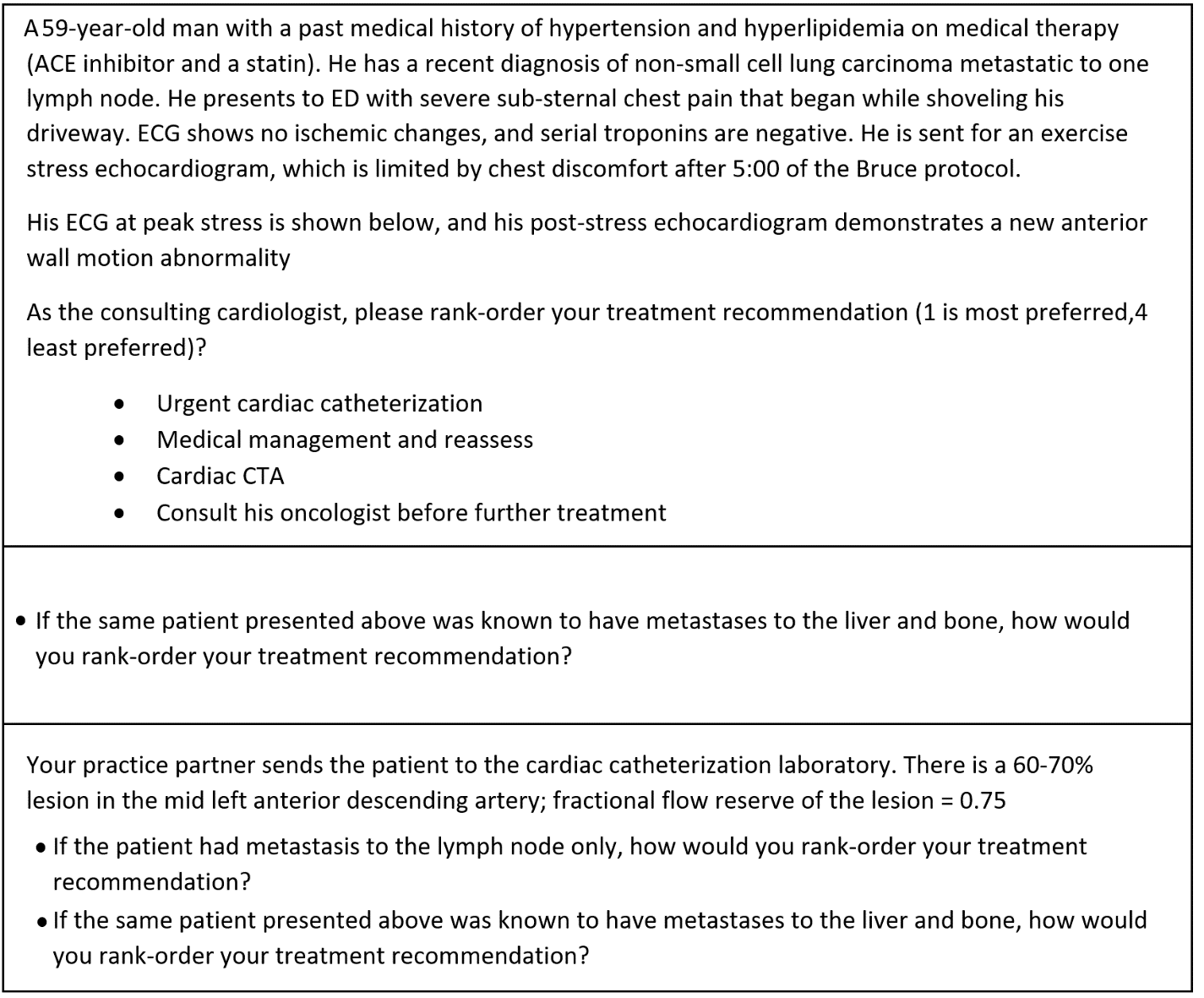

Supplement: Supplemental data [file Suppl_TableS2.docx]
